# Management of Neglected Locked Anterior Dislocation of Shoulder: A Report of Two Cases

**DOI:** 10.7759/cureus.13843

**Published:** 2021-03-12

**Authors:** Rameshwar Datt, Gunjar Jain, Hira Lal Nag

**Affiliations:** 1 Orthopaedics, All India Institute of Medical Sciences, New Delhi, IND

**Keywords:** locked shoulder dislocation, latarjet procedure, neglected shoulder dislocation, bankart surgery

## Abstract

There is a paucity of literature regarding a neglected shoulder dislocation, as it is unusual to miss it clinically due to the apparent deformity. Nevertheless, in some cases, particularly those who received the primary treatment from a local bonesetter, present with neglected dislocation. No high-level studies comparing different treatment modalities in such a situation are available. Therefore, most of the treatment recommendations are based on level four studies and the literature for recurrent dislocation of the shoulder. We herewith describe two cases of neglected anterior dislocation of the shoulder, which we have managed by open reduction and Latarjet procedure in one and Bankart surgery in the other patient. Both of our patients after one-year follow-up had a painless joint with improved yet limited range of motion. This case discussion helps in learning the approach towards the treatment of these patients. It also suggests a sub-optimal functional outcome in them.

## Introduction

The incidence of first-time shoulder dislocation in a UK-based study was 21.9 cases per 1,00,000 population. Out of these, 7.9% sustained another dislocation, and 6.1% suffered persistent symptomatic instability [1]. A neglected anterior dislocation of the shoulder is very rare, as because of the apparent clinical deformity, it rarely goes unnoticed. However, cases that received the primary treatment from a local bonesetter present with neglected dislocation. Elderly patients, chronic alcoholic subjects, and those with a seizure disorder are also at high risk for neglected dislocations [2]. Around 16% of all shoulder dislocations fail to reduce without sedation [3]. These patients may also present late in higher centers, particularly in developing nations, when the primary care center doesn't have facilities for a reduction under anesthesia.

No high-level studies comparing different treatment modalities in neglected dislocation of the shoulder are available. Therefore, most of the treatment recommendations are based on level four studies and the literature for recurrent dislocation of the shoulder. We herewith describe two cases of neglected anterior dislocation of the shoulder, which we have managed by open reduction and Latarjet procedure in one and Bankart surgery in the other patient.

## Case presentation

Case 1

A 38-year-old male patient presented to our outpatient department with chief complaints of pain and restriction of movements of his left shoulder. He was a chronic alcoholic. Six months back, he had a generalized tonic-clonic seizure (GTCS), for which he was managed in a regional hospital, where anti-epileptic medications were started after thorough investigations. However, five months later, due to irregular medications, he suffered from a second GTCS episode. During this GTCS episode, his left shoulder got dislocated. He received the treatment from a local bonesetter, during which he suffered from a fracture of the greater tuberosity, and the dislocation didn't reduce. He has not experienced an episode of seizure since the last month.

**Figure 1:**
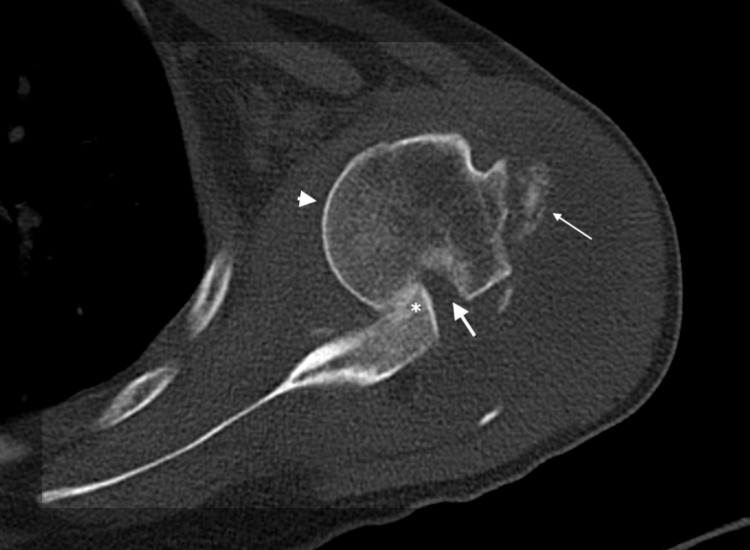
CT scan (axial section) of the left shoulder showing the anteriorly dislocated humeral head (arrow head) locked over the anterior glenoid (asterisk mark) with a large Hill-Sachs lesion (thick arrow) and an associated greater tuberosity fracture (thin arrow).

**Figure 2:**
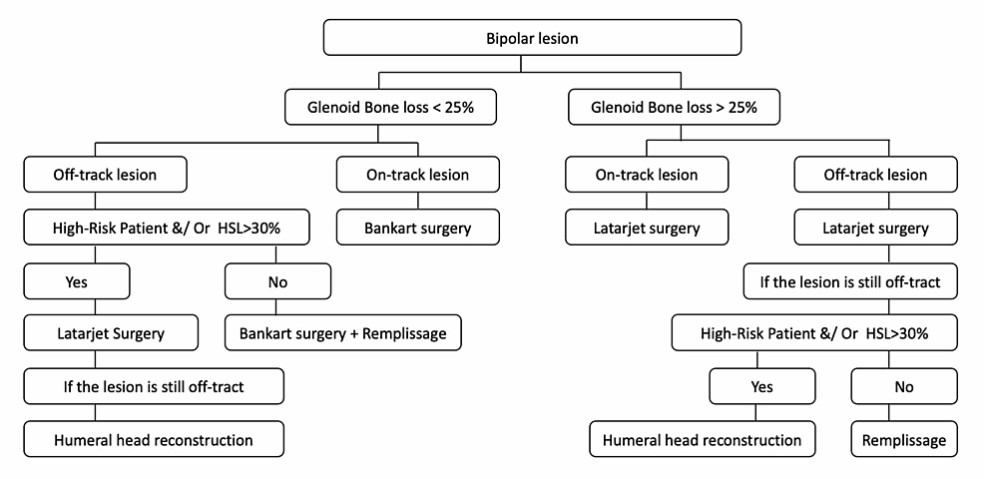
Our treatment protocol for neglected shoulder dislocation with bipolar lesion. Patients with high risk of recurrent dislocations, and those who require a full range of motion or involved in contact sports are included in the high-risk group. HSL: Hill-Sachs Lesion

On physical examination, deltoid muscle wasting was present, the glenoid fossa was empty, the humeral head was palpable in the anteroinferior glenoid region, and the Duga’s test was positive. The range of motion of his left shoulder was painful and grossly restricted. Plain radiography showed anterior dislocation of the glenohumeral joint with associated greater tuberosity fracture. CT scan showed a large engaging Hill-Sachs lesion (HSL) on the humeral head (Figure 1). His glenoid bone loss was 21.1%, and the Hill-Sachs defect occupied 17% of the humeral head. Thus we made a diagnosis of neglected locked anterior shoulder dislocation with off-track HSL. According to our treatment protocol (Figure 2), we planned an open reduction of the dislocation and Latarjet procedure.

Under general anesthesia, under all aseptic precautions, we positioned the patient in a beach chair position. We used the standard deltopectoral approach for the shoulder. We exposed the coracoid and released the pectoralis minor muscle attached medially. Laterally we incised the coracoacromial ligament, leaving a cuff of 1 cm. We did the coracoid osteotomy at the knee of the coracoid. We split the subscapularis muscle at its superior two-thirds and inferior one-third junction and did the capsulotomy. We then reduced the humeral head in the glenoid fossa. Finally, we fixed the coracoid over the anteroinferior glenoid with two cannulated cancellous screws. Following the procedure, the off-track HSL became on-track.

Postoperatively left upper limb was kept in a sling for two weeks. Pendulum exercise was begun immediately. After three weeks, abduction was allowed up to 90 degrees, and external rotation was allowed up to 25 degrees. This exercise program was continued for up to six weeks. Biceps strengthening exercise was restricted until six weeks to protect the coracoid graft. Meanwhile, the antiepileptic medications were continued as per the opinion of the neurology department. The patient has not experienced any further episodes of convulsion.

At three months, the bone graft demonstrated early consolidation, and after that the patient was allowed to perform light work. After one year of the surgery, the patient had achieved forward flexion up to 140 and abduction and external rotation 70 degrees, and his constant score was 72. He has not experienced any episode of dislocation since the surgery.

Case 2

A 56-year-old male patient presented to our outpatient department with chief complaints of pain and restriction of movement of right shoulder for the last six weeks. He had a history of multiple shoulder dislocation. The first dislocation occurred following an event of trauma 10 years back which was reduced in a nearby hospital. He had suffered from three more dislocations since then, each reduced in a hospital. The last episode of dislocation occurred one month back, following a fall onto the right shoulder. This time the patient went to a bonesetter, who attempted to reduce the shoulder but failed. The patient neglected the dislocation and has not sought any further treatment before reporting to us.

**Figure 3:**
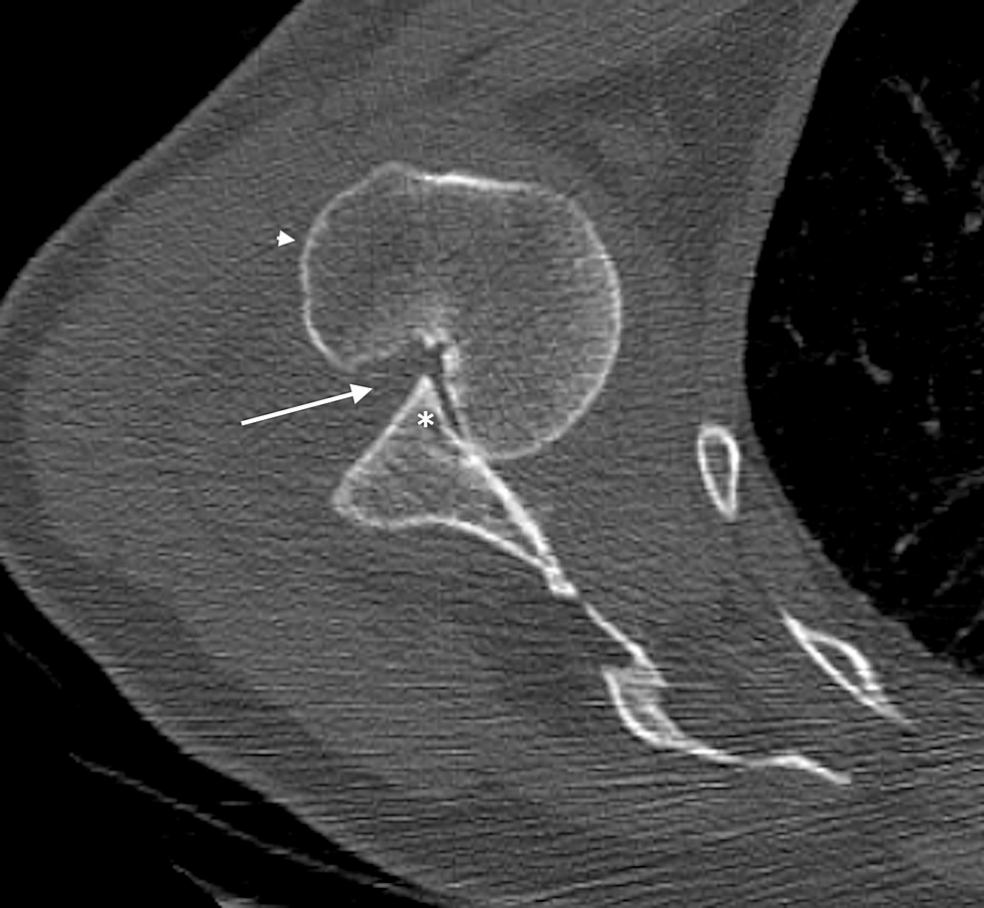
CT scan (axial section) of the left shoulder showing the anteriorly dislocated humeral head (arrow head) locked over the anterior glenoid (asterisk mark) with a large Hill-Sachs lesion (white arrow).

On physical examination, there was a loss of deltoid contour. There was no sensory deficit. Duga’s test was positive. The range of motion of the right shoulder was grossly restricted and painful. His abduction was possible up to 30 degrees, and rotational movements were limited. The constant score in the preoperative period was 37. X-ray imaging showed anterior glenohumeral dislocation, while the MRI was suggestive of a Bankart lesion. CT scan showed a glenoid bone loss of 7% with a non-engaging HSL involving 21% of humeral head (Figure 3). We planned for open reduction and Bankart repair, as per the treatment protocol described earlier.

The patient was positioned supine. Under general anesthesia, after proper draping and painting of the limb, a deltopectoral approach was used to open the joint. The clavipectoral fascia was incised, and the long head of the biceps tendon was traced proximally to identify the rotator interval. We performed lesser tubercle osteotomy and reflected the subscapularis medially to expose the joint capsule. The anteroinferior labrum was found to be torn. After capsulotomy, the joint was reduced, and labral repair was done using two double-loaded metallic suture anchors.

We started pendulum exercise on the first postoperative day and advised the patient to use an arm sling for four weeks. The patient was allowed to do a progressive range of motion exercise with gradual increments. However, we restricted external rotation in abducted position and internal rotation up to six weeks to protect the Bankart repair and the lesser trochanter osteotomy. The patient had achieved a forward flexion of 110 degrees and external rotation of 30-degrees in a neutral position at four weeks, after which we started light theraband exercise. Following six weeks, we initiated the restricted rotational movements and allowed forward flexion up to 130°. At three months, we allowed abduction and external rotation as tolerated without pain and started the theraband exercises against resistance. Finally, we permitted overhead head activities after four months of the surgery. At the end of the one-year, he has maintained the range of forward flexion of 130°. However, he had an abduction of 120° and external rotation of 30°. His constant score was 78 at the final follow-up.

## Discussion

A neglected dislocation is a challenging condition, as the soft tissue contracture around the joint, fibrosis in the glenoid fossa, and bony lesions in both glenoid and humerus complicate the management. Also, as the dislocation is commonly locked, it is unlikely to achieve a closed reduction in such cases. Furthermore, attempting the latter may cause a fracture, as occurred in one of our patients, since most of these patients are elderly, and there is disuse osteoporosis of the bone. However, Raptis et al. had reported a case of four weeks old neglected bilateral locked anterior shoulder dislocation with greater tuberosity fracture on both sides, which they managed non-operatively by closed reduction and gradual mobilization [4]. They had achieved bony union within three months and enough range of motion required for routine activities by one year. Chaudhary et al. had also reported a successful closed reduction of a six months old neglected dislocation [5].

Though most of the neglected dislocations are posterior, chronic locked anterior dislocations are more common than locked posterior dislocations [2]. Both patients in the current case series reported late as they were treated primarily by a local bonesetter. In previous reports, the causes of seeking a delayed treatment by patients with anterior shoulder dislocation include uncontrolled seizure, lack of pain, and negligence by the local physician [5,6].

**Figure 4:**
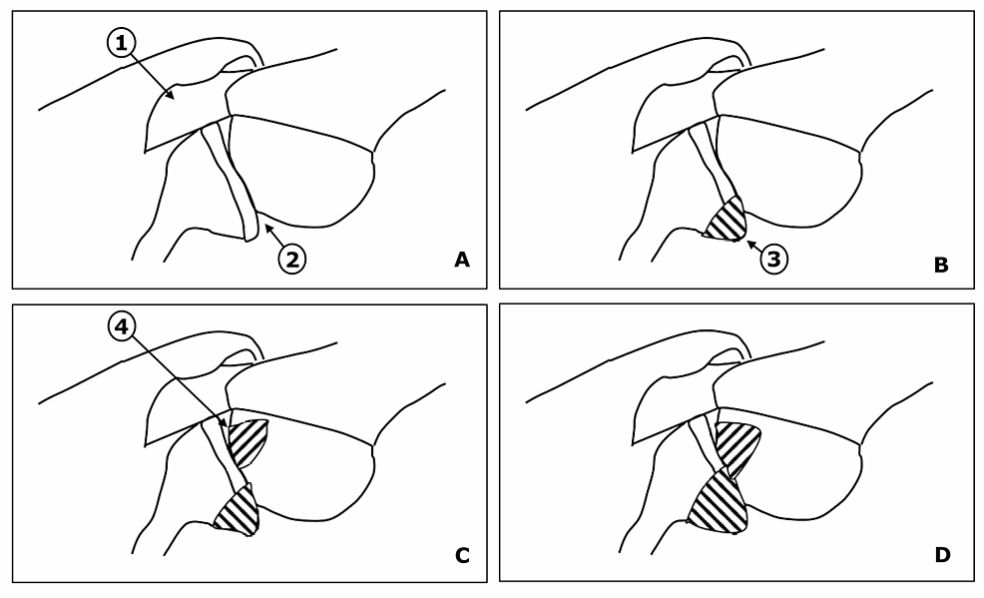
Schematic diagram to demonstrate on-track and off-track Hill-Sachs lesions. (A) A normal shoulder joint in abduction and external rotation position. (B) A shoulder joint with isolated glenoid bone loss. (C) A shoulder joint with an on-track Hill-Sachs lesion, where some portions of the humeral head and glenoid cartilage are still in contact. (D) A shoulder joint with off-track Hill-Sachs lesion, where the medial margin of the Hill-Sachs lesion crosses the anterior margin of the glenoid. Note the presence of a bony bridge between the Hill-Sachs lesion and the medial margin of the rotator cuff muscle insertion over the greater tuberosity. 1: Rotator cuff musculature, 2: Glenoid-humeral joint, 3: Glenoid bone loss, 4: Hill-Sachs lesion

The management of neglected shoulder dislocation depends on multiple factors including, the amount of glenoid and humeral bone loss and the patient's activity level. The 3D-CT examination is the most reliable method to calculate bone loss [7]. Comparison with the contralateral shoulder or the best-fit circle method is commonly used to calculate the percentage of glenoid bone loss [7]. The percentage arc method is usually applied to determine the Hill-Sachs defect size [8]. However, the more critical aspect of the HSL is to determine if the lesion is either on-track or off-track (Figure 4). In an off-track lesion, the medial margin of the humeral defect crosses the anterior border of the glenoid in the abduction external position [7]. Thereby it engages with the glenoid and causes instability.

Patients with a non-critical glenoid bone loss and an engaging HSL with either a high risk of recurrence or more than 30% Hill-Sachs defect can be managed by Latarjet surgery [7,8]. The first patient of the current series had a 21.1% glenoid bone loss, and his humeral head bone loss was 17%, which was off-track. Because of epilepsy, the patient had a higher chance of recurrence [9]. Therefore, we decided to perform a Latarjet surgery for this patient. Both Chaudhary et al. and Peshin et al. had reported similar cases, which they had managed by Latarjet surgery [5,6]. Since, after the coracoid transfer, the HSL was not engaging with the glenoid, we did not perform any additional procedure to address the HSL. Therefore, the necessity for the reconstruction of the HSL should be analyzed after completion of the Latarjet surgery. However, Peshin et al. have performed the autograft reconstruction of the HSL before the coracoid transfer [6].

Our second patient had a bipolar lesion with 7.7% glenoid bone loss and a non-engaging Hill-Sachs defect involving 21% of the humeral head. We managed him by isolated open Bankart surgery as an additional procedure is not indicated in patients with non-engaging HSL and less than 25% glenoid bone loss. Rouhani and Navali have reported the results of open reduction and capsulolabral repair in eight such patients with non-engaging HSL [10]. Although all of their patients were having a loss of motion in every plane, they performed their daily pursuits, and none had significant pain. Our patient was also satisfied with the procedure despite the residual stiffness of the joint.

Patients with subcritical glenoid bone loss and engaging Hill-Sachs lesion can be managed by either a Latarjet procedure or an arthroscopic Bankart repair with remplissage. Both Haroun et al. and Cho et al. found that patients treated by Latarjet surgery had higher post-operative complications like coracoid non-union, screw back out, and osteoarthritis than remplissage group [11,12]. Yang et al. suggested in a prospective cohort study that in the revision scenario patients, who underwent remplissage, had a higher reoperation rate due to residual pain [13]. Itoi had advised that patients involved in contact sports and those who require a full range of motion should be operated on by a Latarjet procedure [7]. We believe that patients with a higher risk of recurrence and those with an HSL involving more than 30% of the humeral head should be operated on by a Latarjet surgery, and rest patients can be treated by Bankart surgery.

## Conclusions

From this case report, we can conclude that the management of patients with a neglected shoulder dislocation depends on multiple factors. These factors include the size of glenoid bone loss and the position of the Hill-Sachs defect. The requirement of the patient and the risk of recurrent dislocation should also be considered. The prognosis in terms of functional outcome is unfavorable in these patients compared to patients with recurrent dislocation.
